# School environment and obesity in adolescents from a Brazilian metropolis: cross-sectional study

**DOI:** 10.1186/s12889-022-13592-0

**Published:** 2022-06-20

**Authors:** Maíra Macário de Assis, Lucia Helena Almeida Gratão, Thales Philipe Rodrigues da Silva, Nayhanne Gomes Cordeiro, Ariene Silva do Carmo, Cristiane de Freitas Cunha, Tatiana Resende Prado Rangel de Oliveira, Luana Lara Rocha, Larissa Loures Mendes

**Affiliations:** 1 Ph.D in Health Sciences, Medical School, Universidade Federal de Minas Gerais, Belo Horizonte, Brazil; 2 Departamento de Enfermagem Materno Infantil E Saúde Pública, Postdoctoral Fellow, Ph.D in Health Sciences, Child and Adolescent Health. Escola de Enfermagem, Programa de Pós-Graduação Em Enfermagem, Universidade Federal de Minas Gerais, MG Belo Horizonte, Brazil; 3 Master in Nutrition and Health, Nutrition Department, Universidade Federal de Minas Gerais, Belo Horizonte, Brazil; 4 PPh.D Health Sciences, Grupo de Estudos, Pesquisas e Práticas em Ambiente Alimentar e Saúde – GEPPAAS, Universidade Federal de Minas Gerais, Belo Horizonte, Brazil; 5 Ph.D in Health Sciences, Pontifícia Universidade Católica de Minas Gerais, Belo Horizonte, Brazil; 6 Ph.D in Public Health, Departamento de Medicina Preventiva E Social, Universidade Federal de Minas Gerais, Belo Horizonte, Brazil; 7 Nursing School, Nutrition Department, Belo Horizonte, MG, Brazil, Universidade Federal de Minas Gerais, Avenida Professor Alfredo Balena, 190 (Escola de Enfermagem), Santa Efigênia, MG 30130100 Belo Horizonte, Brasil

**Keywords:** Adolescents, Obesity, Food Environment, Schools

## Abstract

**Background:**

Childhood-juvenile obesity is a globally acknowledged public health issue. The school environment has been widely assessed because it is where adolescents stay longer during the day, and it may have impact on obesity. School became a crucial environment for obesity prevention in children and adolescents. The aim of the present study was to associate schools’ internal environment factors and its surrounding areas with obesity in adolescents from a Brazilian metropolis.

**Methods:**

Cross-sectional study based on data from the Study on Cardiovascular Risk in Adolescents. The sample comprised 2,530 adolescents in the age group 12–17 years, who were enrolled in public and private schools in Belo Horizonte City, Brazil. Obesity was the dependent variable based on the cut-off point score-z + 2 for body mass index based on age. School environment’s independent variables were ‘managerial dependence type’, ‘number of drinking fountains’, ‘school sports environment’ and ‘ready-to-eat food shops’ around the school (within an 800 m buffer).

**Results:**

Obesity prevailed in 7.21% in sample. The largest number of drinking fountains decrease by 9% the chances of obesity in adolescents enrolled in public and private schools; however, the second and third terciles recorded for the number of ready-to-eat food shops within the 800 m buffer around schools increased by 24% and 44% the chances of obesity, respectively.

**Conclusion:**

School food environment aspects such as the number of operational drinking fountains and the availability of ready-to-eat food shops around the school were associated with obesity in adolescents from a Brazilian metropolis.

## Background

Obesity in adolescents is a globally acknowledged public health issue; moreover, obesity prevalence in adolescents living in low- and middle-income countries rose fast in the last three decades [1]. The 2009 Food and Nutrition Surveillance System has shown that 18.17% of adolescents in the age group 10–20 years in Brazil were overweight, 7.91% were obese and 1.70% were diagnosed with severe obesity [2]. Early obesity, at individual level, can last throughout adulthood and cause other diseases, such as diabetes, high blood pressure and metabolic syndrome, a fact that worsens quality of life. From governments’ viewpoint, the scenario can overburden health care facilities and increase expenses with health care assistance [3].

Environmental factors can stand out among obesity causes, from an ecological perspective [4–7]; and school, where adolescents spend much of their time, is among them [8]. Thus, depending on its features, the school environment can expose adolescents to unhealthy diets [9–12] and discourage exercising(PA) [13]. This process may derive from ultra-processed food sales [12] in school facilities and in their surroundings, as well as from lack of infrastructure for sports and leisure [13].

A systematic review and meta-analysis of 20 studies on the potential association between the school environment and obesity in adolescents showed positive correlation between food sales in school facilities or in their surroundings and BMI, negative association between healthy food availability at school and obesity odds, and null association between the presence of nutrition policies or programs at school and obesity [12]. It was shown that eight out of the twenty reviewed studies did not apply cluster analysis [12]. Dighe et al. (2020) [13] conducted a cross-sectional study and estimated the association between school environment food aspects and PA with BMI outcome in a sample of 6–19-year-old students from public schools in low-income cities in New Jersey (USA) [13]. The aforementioned authors found reversed association between healthier food at school and PA settings, mainly when it comes to healthier options of school food and of fewer unhealthy food, competitive food offers and a larger number of PA facilities and BMI among school-aged children. However, the results found depict adolescents from schools with very minority populations [13].

The aim of the present study was to evaluate adolescents in public and private schools from a Brazilian metropolis to estimate the association between schools’ internal environment factors and their surrounding areas with obesity based on contextual analysis.

## Methods

### Study design, site and duration

Cross-sectional study based on data provided by the Study on Cardiovascular Risks in Adolescents, also known as ERICA, which was a school-based national cross-sectional study conducted from March 2013 to December 2014, with 74,589 adolescents in the age group 12–17 years who were enrolled in public and private schools from a Brazilian metropolis. Detailed information about sample definition, sampling process, research protocol, participants’ selection and data collection at ERICA were published in previous studies [14, 15].

### Research sample

Research sample included adolescents from 1,247 schools, in 124 counties, who were enrolled in the last three years of elementary school and in the three years of high school in 124 counties. The selected counties were divided into 32 geographic strata, namely: 26 state capitals, 1 Federal District and 5 strata representing counties with more than 100 thousand inhabitants located in the country’s macro-regions.

Schools were selected based on both number of students and probability inversely proportional to distance between the state capital and the other counties. Three classes were selected per school, based on different school-shift combinations (morning and afternoon) and grades (7th, 8th and 9th grade of elementary school; first, second and third grade of high school), at equal probability. All students from the selected classes were invited to participate in the study. Adolescents out of the age group 12 to 17 years were excluded from the study, as well as students with some degree of inability capable of impairing their anthropometric evaluation and of stopping them from fulfilling the questionnaire, as well as pregnant adolescents.

Only adolescents enrolled in schools from Belo Horizonte City, capital of Minas Gerais State, were assessed in the current study (2,530 adolescents).

The study was approved by the Ethics Committee in Research of the institution belonging to the study’s central coordination (IESC/UFRJ) and of each Brazilian state. The cut-off point set for Belo Horizonte, in the current study, was approved by the Research Ethics Committee of Federal University of Minas Gerais (CAAE: 61,335,316.5.0000.5149; Opinion 3.691.415 and CAAE: 38,003,220.4.0000.5149; Opinion 4.454.467). All adolescents who have participated in the study signed the consent form and their parents, or legal guardians, signed the free and informed consent form.

### Study protocol

#### Outcome variable

Anthropometric measurements of the full sample were taken by trained researchers. Weight was measured through single measurement on electronic scale Líder® (200 kg capacity and 50 g variation) and height was calculated based on the mean of two measurements taken in sequence, in portable and detachable stadiometer (Alturexata®), at millimeter resolution and at up to 213 cm field of use.

#### Individual explanatory variables and adjusted variables

Data of each participant were collected through a questionnaire that was answered by the participants in electronic device (PDA, *Personal Digital Assistants*) – there was no intervention by researchers.

The quantitative variable ‘number of operational drinking fountains” was found based on the following questions: “Are there drinking fountains in the school?” and “How many of them are working?”, which were answered by school principals.

The proxy school-environment variable for exercising comprised variables “outdoor courtyard”, “indoor courtyard”, “pool” and “sports availability after school shift”, which were built through Principal Component Analysis (PCA). Variables used in this composition were provided by school principals, who had provided information on the number of sports spaces within the questionnaire (outdoor courtyard, indoor courtyard and pool), as well as on sports activities provided by the school to students after the school shift (wrestling, soccer, volleyball, swimming and athletics). Two categories were taken into consideration, namely: “less favorable conditions” and “more favorable conditions” for exercising. The “more favorable conditions” category was featured by the largest number of indoor courtyards, larger number of sports modalities offered by the school (wrestling, soccer, volleyball, swimming and athletics, among others) and having a pool in the school.

Variable “how often legal guardians have supper with the adolescent” was measured through the question: “Does your father (or step-father) or mother (or step-mother), or legal guardian, have supper with you?”—the following answers were available: “almost never”’, “sometimes”, “almost every day” and “every day”. Variable “living with parents” was calculated by combining variables “living with the mother (yes/no)” and “living with the father (yes/no)”. Variable “having breakfast” was calculated based on the question: “Do you have breakfast?”. Answer options were “I do not have breakfast”; “I have breakfast sometimes”; “I have breakfast almost every day” and “I have breakfast every day”. Exercising was classified as inactive (0 mn/week), insufficiently active (1 – 299 min/week) and active (≥ 300 min/week), according to WHO’s recommendations [16].

The socioeconomic score was assessed in ERICA based on questions about owning assets and comfort items according to the social stratification of the Brazilian Economic Classification Criteria (CCEB), which was elaborated by the Brazilian Association of Research Companies [17]. However, no information about mother’s schooling was provided in 20.41% of questionnaires. Thus, by taking into account that the exclusion of these individuals would represent significant loss for the sample, the option was made to only sum the score recorded for “owning assets” to “having a housekeeper” (which was renamed as socioeconomic score in the current study). The socioeconomic score was featured based on three equal intervals, as defined by Moura et al., [18], namely: low socioeconomic level, 0 -12; medium socioeconomic level, 13–25; and high socioeconomic level, 26–38.

#### Geographical data featuring

School address was provided by ERICA’s central committee so it would be possible assessing the food environment around the school. Information on food sales shops from 2014 was provided by the Sub-secretariat of State Revenue, Superintendence of Tax Collection and Information, and by the Direction of Economic-Fiscal Information of Minas Gerais State Government. The list by the National Classification of Economic Activities (CNAE) was used to feature these shops based on their main activity [19].

Schools and food shops were georeferenced based on latitude and longitude information processed in the QGIS 2.8.6 and ArcGis 10.8 software to determine the number of food sales shops around the school. A 800 m buffer was drawn from the school’s centroid [20]. Collected data were inserted in the sampled adolescents’ database – a single database number was created.

The analyzed food sales shops around the assessed schools were the ones selling ready-to-eat food, as suggested by Peres et al. [9]. The following shop categories were taken into consideration: dinners, snack shops, bars, restaurants, bakeries, supermarkets, hypermarkets and grocery stores.

The VHI of schools’ vicinities is one of the synthetic measurements offset for socioeconomic and sanitation conditions; it was built from information provided by the 2010 demographic census on income, education, work, leisure and social insertion. This index ranges from 0 to 1—the higher its value, the greater the health vulnerability of the census sector [21].

### Statistical analysis

#### Outcome variable

Obesity was the adopted dependent variable, which was classified based on adolescents’ weight and height measurements. The score-z + 2 was the adopted cut-off point for body mass index, based on age [22].

#### Independent variables

Environmental independent variables were ‘school managerial dependence type (public/private)’, ‘number of operational drinking fountains in the school”, school sports environment’ and ‘food sales environment around the school’—which was assessed through ready-to-eat food shops within a 800 m buffer around the school.

#### Adjustment variable

The following adjustment variables were included in the study: sex (male/female), age group (< 15 years/ ≥ 15 years), self-referred skin color (white, black or brown, Asian, Indigenous), socioeconomic score (high, medium or low), living with parents (with both parents, only with the mother or with the father, does not live with the parents), how often the legal guardian has supper with the adolescent (never or almost never, sometimes, almost every day, every day), how often the adolescent has breakfast (does not have it, sometimes, almost every day, every day), exercising (inactive, insufficiently active, active), and school surroundings’ vulnerability for health index (VHI) (low, medium, high and very high risk).

The descriptive analysis of individual variables and of schools’ internal and external environment was carried out by calculating frequencies’ distribution through the survey modulus to analyze data of a complex sample.

The principal component analysis (PCA) was performed to create the school environment variable for exercising. Variables ‘outdoor courtyard’ and ‘sports modality offered (wrestling, soccer, volleyball, swimming and athletics) after school shift’, and the number of pools in the school were included in the PCA. *Kaiser-Mayer-Olkin* (KMO) coefficient was estimated as PCA adjustment measurement, at values ranging from 0.5 to 1.0, which were considered acceptable for this index. Components recording eigenvalues > 1.0—defined according to the scree plot graph, were extracted from the PCA. Components’ structure was found through variables presenting factorial load higher than 0.3—a variable within the score units was generated for each school environment category. These variables enabled creating a new categorical variable, which was divided by the median of the score distribution recorded for this category.

### Multilevel analysis

Association between school internal and external environment variables and obesity in adolescents enrolled in public and private schools was estimated through the multilevel logistic models. The bivariate analysis was carried out through multilevel logistic regression model, which used obesity as dependent variable. Variables showing *p*-value < 0.20 in the bivariate analysis were used as covariates in the multivariate model.

Four models were proposed for the multivariate analysis: i. empty model; ii. only individual variables; iii. only environmental variables; and iv; individual and environmental variables. The Intraclass Correlation Coefficient (ICC) was estimated to quantify the obesity:explained variance ratio at individual and contextual level. The model’s adjustment evaluation was performed by comparing Akaike’s Information Criterion (AIC) values, since AIC value reduction points towards better model adjustment for the response variable.

The statistical package of the *Statistical Software for professional* (STATA), version 14.0, was used for the calculations. The multilevel models used the gllamm command, which works with non-independent data and with multilevel analysis, based on the inclusion of sampling weighs for complex samples. The analyses took into consideration individuals as level 1 unit and schools as level 2 unit. *Odds ratio* (OR) at 95% confidence interval (95% CI) was used to measure the association. Significance level was set at 5%.

### Ethics approval and consent to participate in the study

The study was approved by the Research Ethics Committees of the institution coordinating the study (IESC/UFRJ) and of each Brazilian state. Adolescents who agreed to participate in the study signed the written informed consent form; parents or legal guardians signed the written informed consent form of all under-18 participants. Participants’ identification remained confidential.

## Results

Based on the analyses, 2,530 adolescents were in the age group 12–17 years and enrolled in 43 schools in Belo Horizonte City, Minas Gerais State, Brazil. Obesity prevailed in 7.21% (CI 95% 6.13 – 8.47) of the sample. According to the descriptive analyses, most adolescents belonged to the male sex (50.32%), they were younger than 15 years (51.21%) and declared themselves as black or brown (62.53%) (Table 1). It was also possible observing that only 1.28% of them have presented low socioeconomic score and 4.5% did not live with their parents. Adolescents who have reported to never, or almost never, have supper with their legal guardians, and who do not have breakfast, reached 13.23% and 17.48%, respectively. Furthermore, 30.84% of them were classified as physically inactive (0 min./week) (Table 1).

**Table 1:** Sample featuring based on obesity in students in the age group 12–17, from Belo Horizonte City, Minas Gerais State, Brazil, 2013 -2014

Variable	Total	Obese	Non-obese	Non-adjusted OR *	CI 95%		*P*-value
	**%**	**%**	**%**				
Sex (*n* = 2.530)							
Male	50.32	50.08	50.34	Ref			
Female	49.68	49.92	49.66	1.04	0.95	1.15	0.352
Age group (*n* = 2.530)							
< 15 years	51.21	63.82	50.23	Ref			
> = 15 years	48.79	36.18	49.77	0.67	0.6	0.76	< 0.001*
Self-referred skin color (*n* = 2.473)							
White	33.8	24.51	34.49	Ref			
Black or brown	62.53	71.19	61.89	2.21	1.95	2.5	< 0.001*
Asian	2.31	3.18	2.25	1.21	0.78	1.87	0.389
Indigenous	1.36	1.12	1.37	1.22	0.71	2.09	0.454
Socioeconomic score (*n* = 2.473)							
High	29.52	37.44	28.94	Ref			
Medium	69.2	61.24	69.79	0.73	0.65	0.81	< 0.001*
Low	1.27	1.32	1.28	0.75	0.49	1.15	0.193*
Lives with parents (*n* = 2.530)							
Both parents	56.65	61.04	56.31	Ref			
Only with the mother or the father	38.81	33.02	39.26	0.78	0.7	0.87	< 0.001*
No	4.54	5.94	4.43	2.01	1.67	2.42	< 0.001*
How often the legal guardian has supper with the adolescent (*n* = 2,530)							
Never or almost never	13.23	17.41	12.9	Ref			
Sometimes	24.14	26.77	23.93	0.78	0.67	0.9	< 0.01*
Almost every day	21.04	17.47	21.31	0.48	0.41	0.57	< 0.001*
Every day	41.59	38.35	41.86	0.54	0.47	0.62	< 0.001*
Breakfast-having frequency (*n* = 2,530)							
Does not have it	17.48	20.1	17.28	Ref			
Sometimes	27.31	33.76	26.81	0.89	0.78	1.02	0.124*
Almost every day	12.59	11.17	12.7	0.56	0.46	0.67	< 0.001*
Every day	42.62	34.97	43.21	0.48	0.42	0.55	< 0.001*
Exercising (*n* = 2,530)							
Inactive (0 min/week)	30.84	34.71	30.54	Ref			
Insufficiently active (1 – 299 min/week)	27.18	27.79	27.13	0.85	0.75	0.96	0.01*
Active (≥ 300 min/week)	41.98	37.50	42.33	0.86	0.77	0.97	0.014*

Weighed sample rate. * *p* < 0.20

Table 2 presents the PCA results, and the component loads of variables forming the school sports environment component, which is featured by having an outdoor courtyard, availability of different sports modalities after the school shift and by the largest number of pools in the schools (Table 2).

**Table 2:** Component loads formed by principal component variables related to exercising in school environments included in ERICA study. Brazil, 2013–2014

Indicator	Component	KMO
Outdoor courtyard	**0.6560**	0.5301
Sports modality availability after school shift	**0.3242**	0.5685
Number of pools	**0.6815**	0.5525
*Eigenvalue*	1.72	-
*Explained variance (%)*	57.18	-
*General*	-	0.5247

*KMO* Kaiser–Meyer–Olkin

In total, 77.58% of the schools were public, 64.63% of them had unfavorable school sports environment and their mean number of drinking fountains was 6.34 (SD = 0.52). Yet, 48.95% of the sample comprised adolescents enrolled in schools located in neighborhoods accounting for low VHI—26.22% of schools were in the highest tercile recorded for the number of ready-to-eat food shops within the 800-m buffer around the school (Table 3).

**Table 3:** Featuring the school environment based on obesity in students in the age group 12–17 years from Belo Horizonte City, Minas Gerais State, Brazil, 2013 -2014

Variables	Total	Obese	Non-obese	Non-adjusted OR	CI 95%		*P*-value
	**%**	**%**	**%**				
School environment for exercising ^a^ (*n* = 2,530)							
Unfavorable conditions	64.63	64.19	64.61	Ref			
More favorable conditions	35.37	35.81	35.39	0.76	0.66	0.89	< 0.001*
Vulnerability to health index (VHI) (*n* = 2,466)							
Low	48.94	49.86	48.87	Ref			
Medium	43.62	43.2	43.65	0.83	0.71	0.97	0.021*
High and very high	7.44	6.94	7.48	0.7	0.46	1.07	0.106
School managerial dependence type (*n* = 2,530)							
Public	77.58	77.76	77.57	Ref			
Private	22.42	22.24	22.43	0.55	0.45	0.66	< 0.001*
Ready-to-eat food shops within the 800-m buffer around the school ^b^ (*n* = 2.530) (min., max.)							
1st tercile (2 – 49)	37.78	33.91	38.07	Ref			
2nd tercile (50 – 79)	36	43.7	35.41	1.25	1.05	1.49	0.011*
3rd tercile (85 – 437)	26.22	22.39	26.52	1.23	1.04	1.46	0.015*
Number of drinking fountains ^c^	6.34 (0.52)	5.66 (0.49)	6.39 (0.53)	0.93	0.91	0.94	< 0.001*

Weighed sample rate. *p < 0.05

^a^ Most favorable conditions: larger number of indoor courtyards, larger number of sports modality available at school (wrestling, soccer, volleyball, swimming and athletics, among others) and availability of pools in school environment

^b^ Dinners, snack shops, bars, restaurants, supermarkets, hypermarkets and grocery stores

^c^ Variable ‘number of drinking fountains’ is quantitative and it is expressed in means (standard deviation)

Table 4 presents four models: one null and three adjusted models. Based on models 1 to 4, the increase in the number of operational drinking fountains (OR = 0.91; CI95% 0.89 – 0.93) has significantly decreased the chances of obesity (*p* < 0.05) after adjustments were done in the models for individual variables. The second (OR = 1.24; CI95% 1.06 – 1.44) and third (OR = 1.44; IC95% 1.17 – 1.77) terciles recorded for the number of ready-to-eat food shops around the school (800 m) have increased the chances for obesity (*p* < 0.05). The sports environment did not present statistically significant association. It was possible observing AIC reduction in the null model after the individual and school-level variables were included in all models.

**Table 4:** Multilevel logistic regression models set for internal and external school environment factors associated with obesity in adolescents enrolled in schools from Belo Horizonte City, Minas Gerais State, Brazil—2013–2014

Variable	Null Model			Model 1			Model 2			Model 3		
	**OR**	**CI 95%**		**OR**	**CI 95%**		**OR**	**CI 95%**		**OR**	**CI 95%**	
**INDIVIDUAL VARIABLES**												
**Sex**												
Male				Ref						Ref		
Female	-	-	-	0.98	0.88	1.09	-	-	-	0.96	0.86	1.07
**Age**												
< 15 years				Ref						Ref		
> = 15 years	-	-	-	0.72*	**0.63**	**0.81**	-	-	-	0.65*	**0.58**	**0.73**
**Socioeconomic score**												
High				Ref						Ref		
Medium	-	-	-	0.72*	**0.64**	**0.80**	-	-	-	0.76*	**0.68**	**0.86**
Low	-	-	-	0.75	0.49	1.15	-	-	-	0.74	0.48	1.15
**Lives with the parents**												
Both				Ref						Ref		
Only with the mother or father	-	-	-	0.80*	**0.71**	**0.90**	-	-	-	0.71*	**0.63**	**0.80**
No	-	-	-	2.00*	**1.65**	**2.43**	-	-	-	1.86*	**1.52**	**2.27**
**How often the legal guardian has supper with the adolescent**												
Never or almost never				Ref						Ref		
Sometimes	-	-	-	0.72*	**0.61**	**0.85**	-	-	-	0.65*	**0.55**	**0.77**
Almost every day	-	-	-	0.66*	**0.55**	**0.78**	-	-	-	0.60*	**0.50**	**0.71**
Every day	-	-	-	0.70*	**0.60**	**0.82**	-	-	-	0.61*	**0.52**	**0.71**
**Breakfast-having frequency**												
Does not have it				Ref						Ref		
Sometimes	-	-	-	0.98	0.84	1.13	-	-	-	0.97	0.83	1.13
Almost every day	-	-	-	0.69*	**0.57**	**0.84**	-	-	-	0.73*	**0.60**	**0.89**
Every day	-	-	-	0.49*	**0.42**	**0.57**	-	-	-	0.49*	**0.42**	**0.57**
**Exercising**												
Inactive				Ref								
Insufficiently active	-	-	-	0.86	0.75	0.99	-	-	-	-	-	-
Active	-	-	-	0.88	0.77	1.00	-	-	-	-	-	-
**ENVIRONMENTAL VARIABLES**												
**School sports environment**												
Unfavorable conditions							Ref					
More favorable conditions	-	-	-	-	-	-	1.00	0.85	1.18	-	-	-
**Vulnerability to health index**												
Low							Ref			Ref		
Medium	-	-	-	-	-	-	0.74*	**0.63**	**0.88**	0.99	0.86	1.15
High and very high	-	-	-	-	-	-	0.66*	**0.44**	**0.98**	0.58*	**0.40**	**0.83**
**Managerial dependence**												
Public							Ref			Ref		
Private	-	-	-	-	-	-	0.61*	**0.49**	**0.76**	0.66*	**0.55**	**0.80**
**Ready-to-eat food shops within the 800 m buffer around the school**												
1st tercile							Ref			Ref		
2nd tercile	-	-	-	-	-	-	1.52*	**1.29**	**1.80**	1.24*	**1.06**	**1.44**
3rd tercile	-	-	-	-	-	-	1.74*	**1.38**	**2.17**	1.44*	**1.17**	**1.77**
**Number of operational drinking fountains**	-	-	-	-	-	-	0.90*	**0.87**	**0.92**	0.91*	**0.89**	**0.93**
**Intercept**	0.08	0.07	0.08	0.22	0.18	0.28	0.09	0.07	0.11	0.42	0.32	0.55
*AIC (ICC)*	13,300.20 (0.07)			11,432.64 (0.04)			12,479.02 (0.03)			10,737.15 (0.00)		

*OR* Odds Ratio, *CI95%* 95% Confidence Interval, *Ref.* Reference, *AIC* Akaike's Information Criterion, *ICC* Intraclass Correlation Coefficient

^*^p < 0.05

## Discussion

The herein recorded results have suggested that school environment features, such as number of operational drinking fountains and the availability of ready-to-eat food shops around the school were associated with obesity in public and private school adolescents from a Brazilian metropolis.

The largest number of operational drinking fountains was the school environment factor associated with reduced chances of obesity in adolescents. It is so, because drinking fountains’ availability in school environment can favor water intake by students. According to an intervention study carried out in Los Angeles, USA, drinking water supply in school cafeterias combined to food and nutrition education promotion actions were associated with higher drinking water consumption by adolescents in school environment [23].

Accordingly, water availability at school to replace sugary drinks, 100% fruit juice and other beverages’ intake can, somehow, be a beneficial intervention measure for students’ health. A study performed in New York City, USA, has shown that water availability was associated with weight loss in elementary and high school students [24], because the over consumption of sugary drinks is a risk factor for the development of non-communicable chronical diseases, given their free sugar content [25]. On the other hand, drinking water consumption is related to several benefits to health, such as improved cognition, reduced number of dental cavities, lower body weight, as well as to lower sugary drinks’ intake by children and adolescents, mainly when this action is associated with other healthy behaviors [23, 26, 27].

It is essential emphasizing that WHO acknowledges the importance of ensuring access to drinking water at schools. Along with a series of other recommendations, this orientation is included in two documents issued by WHO about strategic actions focused on reducing sugary drinks’ intake in school environment and on preventing obesity in children and adolescents [28].

It was possible observing that the second and third terciles recorded for ready-to-eat food shops within the 800-m buffer around the school unit were associated with increased chances of obesity. This finding can be explained by the likely impact of unhealthy food sold in these shops, which could have influenced students’ food consumption and, consequently, their overweight/obesity condition [29, 30]. Snack bars and pubs are the prevailing commercial establishments selling ultra-processed food in Brazil [31]. An ecological study conducted in Belo Horizonte, Brazil, found the predominance of these establishments around schools [9], along with restaurants [9, 31].

Adolescents likely exposure to an obesogenic food environment close to the place where they spend much of their time can be harmful, since ultra-processed food has strong appeal to adolescents, since they tend to have greater purchasing autonomy than children, mainly when it comes to buying these food types in stores located near schools [32].

The present study has some limitations; it did not take into account the features of school surroundings, such as the presence of parks, green areas, among others, to encourage sports’ practicing; and it did not used food intake data, since information collected at ERICA did not specify consumption location, a fact that made it impossible defining food intake in school environment. The ‘breakfast-having’ variable was included in the analysis to minimize such limitations, given its importance for the assessed outcome [33]. This study evaluated a Brazilian capital and it cannot be extrapolated to other metropolises.

## Conclusion

Internal and external school food environment aspects such as number of operational drinking fountains and the availability of stores selling ready-to-eat food within the 800 m buffer around the school were associated with obesity in adolescents from a Brazilian metropolis.

## Data Availability

Datasets used and analyzed during the current study can be provided by the corresponding author on reasonable request.

## Abbreviations

ERICA: Study on Cardiovascular Risks in Adolescents
IESC: Ethics Committee in Research
UFRJ: Universidade Federal do Rio de Janeiro
PCA: Principal Component Analysis
CNAE: National Classification of Economic Activities
VHI: School surroundings’ vulnerability to health index
CCEB: Brazilian Economic Classification Criteria
KMO: *Kaiser-Mayer-Olkin*
ICC: Intraclass Correlation Coefficient
AIC: Akaike’s Information Criterion
STATA: *Statistical Software for professional*
OR: *Odds ratio*
